# Classification of clinically actionable genetic mutations in cancer patients

**DOI:** 10.3389/fmolb.2023.1277862

**Published:** 2024-01-11

**Authors:** Muhammad Shahzad, Muhammad Rafi, Wadee Alhalabi, Naz Minaz Ali, Muhammad Shahid Anwar, Sara Jamal, Muskan Barket Ali, Fahad Abdullah Alqurashi

**Affiliations:** ^1^ National University of Computer and Emerging Sciences, Karachi, Pakistan; ^2^ Department of Computer Science, Immersive Virtual Reality Research Group, King Abdulaziz University, Jeddah, Saudi Arabia; ^3^ Department of Computer Science, HECI School, Dar Alhekma University, Jeddah, Saudi Arabia; ^4^ Department of AI and Software, Gachon University, Seongnam-si, Republic of Korea; ^5^ Department of Computer Science, Faculty of Computing and Information Technology, Jeddah, Saudi Arabia

**Keywords:** personalized medicine, genetic mutations, machine learning, natural language processing, precision medicine

## Abstract

Personalized medicine in cancer treatment aims to treat each individual’s cancer tumor uniquely based on the genetic sequence of the cancer patient and is a much more effective approach compared to traditional methods which involve treating each type of cancer in the same, generic manner. However, personalized treatment requires the classification of cancer-related genes once profiled, which is a highly labor-intensive and time-consuming task for pathologists making the adoption of personalized medicine a slow progress worldwide. In this paper, we propose an intelligent multi-class classifier system that uses a combination of Natural Language Processing (NLP) techniques and Machine Learning algorithms to automatically classify clinically actionable genetic mutations using evidence from text-based medical literature. The training data set for the classifier was obtained from the Memorial Sloan Kettering Cancer Center and the Random Forest algorithm was applied with TF-IDF for feature extraction and truncated SVD for dimensionality reduction. The results show that the proposed model outperforms the previous research in terms of accuracy and precision scores, giving an accuracy score of approximately 82%. The system has the potential to revolutionize cancer treatment and lead to significant improvements in cancer therapy.

## 1 Introduction

Cancer remains one of the deadliest diseases worldwide, posing a significant health threat and claiming thousands of lives every year. It is a complex condition that can develop in any part of the body. It is characterized by the uncontrolled and abnormal growth of cells, which can lead to the formation of tumors and the potential invasion of neighboring tissues. In more advanced stages, cancer cells can even spread to distant parts of the body through the bloodstream. Normal body cells grow, divide, and die in a regulated cycle, but cancer cells lose this ability to regulate their growth and division.

Cancer tumors arise from a pattern of changes in the DNA sequence of certain genes, known as genetic mutations. These mutations can occur spontaneously during DNA replication or can be caused by exposure to environmental factors such as chemicals and radiation. The human body is estimated to have approximately 20,000 genes Rocha (2023), and mutations can occur in any of these genes. As a result, a single gene can have many different variations or mutations, which can have different effects on protein function and cellular behavior. Genetic mutations have the potential to affect the function of the protein that the gene encodes. This disruption can interfere with the normal mechanisms that regulate cell growth and division. This may result in the abnormal proliferation of cells leading to the eventual development of a cancer tumor. Interpreting these mutations is essential to distinguish the mutations that contribute to tumor growth called driver mutations, from the harmless mutations known as passenger mutations. Once the genetic mutations that contribute to cancer tumors are narrowed down, diagnosing the type of cancer and designing gene-specific treatment becomes easier. To help classify the different types of genetic mutations, expert oncologists from MSKCC use a system that categorizes mutations into nine different classes. These categories include gain-of-function, loss-of-function, neutral, switch-of-function, and others. Gain-of-function mutations can cause a protein to gain a new or enhanced function, while loss-of-function mutations can cause a protein to lose its normal function or become non-functional. Neutral mutations have no effect on protein function or cellular behavior, while switch-of-function mutations cause a protein to gain a new function while losing its original function. Inconclusive mutations are those for which the effect on protein function or cellular behavior is not clear. The other classes of mutations are likely variants of the gain-of-function, loss-of-function, neutral, and switch-of-function categories, but their effects on protein function are predicted rather than experimentally observed.

For many years, most cancer patients diagnosed with the same specific type and stage of cancer have been treated similarly, based on the assumption that they would respond similarly to the same kinds of treatment. However, such treatments are not necessarily effective for every individual and may even result in adverse side effects in some patients. This is due to the fact that every cancer tumor consists of a combination of thousands of different genetic mutations, and is thus unique in nature Krzyszczyk (2018). This heterogeneity in tumors across different cancer patients causes tumors to respond differently to the same type of treatment depending on their genetic mutations. Hence, traditional methods of cancer therapy are now evolving towards a personalized approach, that takes into account the unique genetic makeup of each patient’s tumor.

Personalized medicine in cancer therapy aims to target each cancer tumor uniquely based on the genetic mutations it contains, and prescribe personalized treatment to each cancer patient tailored specifically to that individual’s cancer tumor. The introduction of personalized medication approaches has made it clear that unique treatments are much more efficient for specific sufferers than for others. Since a cancer tumor may consist of thousands of genetic mutations belonging to different types, interpreting the mutations in a tumor is essential to distinguish the cancer-causing driver mutations, from the silent passenger mutations, so that only the driver mutations can be specifically targeted for treatment. Hence, designing a personalized treatment for a cancer patient requires that the mutations are understood in advance. However, the process of classifying clinically actionable mutations involves a huge amount of manual work as it requires pathologists to review and classify every single genetic mutation after reading and interpreting extensive text-based clinical literature as evidence. This method can be highly time-consuming, laborious and subjective, making classification of genetic mutations prone to error, costly and susceptible to bias. This greatly hinders the adoption of personalized medicine in cancer therapy and makes it futile for emergency cases and time-critical cancer patients. Several natural language processing (NLP) techniques can be employed to process and interpret textual evidence on the basis of which genetic mutations can be classified into their respective types with the help of machine learning and deep learning classification algorithms. This would in turn facilitate oncologists a great deal in the detection and related treatment of cancer tumors in a much more efficient and faster manner compared to the manual approach followed by pathologists Gupta (2021).

Several machine learning and deep learning algorithms have been employed in the past to design classifier models that identify gene mutations based on image or text-based pieces of evidence, and can be used to predict diseases. Research specific to certain types of cancer such as brain tumor and breast cancer has also been done. However, research addressing the classification of clinically actionable genetic mutations based on lengthy, text-based clinical evidence is limited, and the achieved accuracy in existing work falls short of the desired level, making the classifier models difficult to rely on. Most researchers have selected a single classification model in which with large text feature sets are fed as the research articles in the data sets are very lengthy, with some articles even consisting of more than 80,000 words. However, a very limited amount of work uses feature selection and dimensionality reduction techniques to reduce the feature set in order to make the classification process more efficient. Low accuracy scores could be the lack of use of efficient text transformation and dimension reduction models. Xu (2019) uses metrics like extracting the top 2500 TF-IDF features of each ID, sorting them according to importance and then using the top 500 sorted words to replace the texts to reduce the feature set, as the performance of ML models are dependent on feature extraction methods. Other works that use deep learning models and neural networks rely on the model’s automatic acquisition of feature expression capabilities, thus eliminating complex manual feature engineering processes and reducing possible application costs Su (2020).

In our proposed approach, we begin by conducting exploratory data analysis on the selected data set to gain insights and understand the distribution of samples across different classes in the training set. Addressing the challenge of imbalanced sample distribution, we use oversampling and under sampling techniques to achieve a more balanced representation. Next, we utilize Natural Language Processing (NLP) techniques to preprocess the extensive text data from the articles. This involves converting the text into numeric features using the TF-IDF feature extraction method. The Singular Value Decomposition (SVD) feature reduction method is employed to handle the high-dimensional and sparse nature of the TF-IDF matrix. The resulting set of features is then used as input for the Random Forest Classifier Model. To evaluate the performance of our approach, we split the data set into an 80–20 train-test ratio. The obtained results demonstrate an accuracy of 0.82, surpassing the performance of recent models in terms of other metrics such as recall and f1 measure.

To summarize, this research has the following key contributions:

• Development of a novel approach for classifying clinically actionable genetic mutations based on textual clinical evidence.
• Implementation of oversampling and undersampling techniques to address the challenge of imbalanced sample distribution in the dataset.
• Evaluation of the proposed approach on a comprehensive dataset, demonstrating superior accuracy, recall, and f1 measure compared to existing models.
• Contribution to the advancement of personalized medicine by enabling efficient and accurate classification of genetic mutations for tailored cancer treatment strategies.

This paper is structured as follows: Section 2 provides a review of relevant literature, examining previous work in the field. Section 3 presents the materials and methodology employed in this research in detail. The testing and evaluation methods are discussed in Section 4, with the results and corresponding discussion presented in Section 5 and Section 6, respectively. Section 7 explores the limitations of this research, highlights its future scope, and concludes the paper.

## 2 Literature review

Personalized treatment for a cancer patient can be designed if the mutations are understood in advance. In previous researches, several machine learning and deep learning algorithms have been employed to design classifier models that identify gene mutations based on image or text-based pieces of evidence, and can be used to predict diseases. Research specific to certain types of cancer such as brain tumor and breast cancer has also been done. However, research addressing the classification of clinically actionable genetic mutations based on lengthy, text-based clinical evidence is limited, and the achieved accuracy in existing work falls short of the desired level, making the classifier models difficult to rely on. Text evidence has been processed using various natural language processing (NLP) techniques, and fed into a machine learning or deep learning model for classification. Some researchers prefer to use deep neural networks and deep learning (DL) algorithms for text categorization due to their ability to learn features automatically from the data.

For pre-processing of data, Xu (2019) has filtered stop words from text to reduce redundancy. Moreover, floating point numbers were also cleared out from text. In addition to NLTK’s English stop words and punctuation, unimportant keywords by observing the text, for example,: “fig”, “figure”, “table”, “using”, “method”, “result”, “conclusion”, “author”, “find”, “found”, “demonstrate”, etc., are also removed. Gupta (2021) removed a total of 433 unnecessary keywords and stopwords like “mutations”, “mutated”, “cell”, “cells”, “protein” and so on which occurs frequently in every class hence play no significant role in classification. This also reduces the size of the feature set considerably.

For text-based datasets, a conversion of text features, which are categorical data to numeric data is required for any classification algorithm to be adopted. Various methods like the bag-of-words model, term frequency–inverse document frequency (tf-idf) scheme or Word2vec could be used for this purpose, tf-idf being the most popular in recent studies due to its ability to assign scores to words considering both; their frequencies in a document and across documents in corpus. In TfidfVectorizer, the overall weightage of a word occurring in a document is considered Bruno (2019). Through this, we can penalize the words that occur most frequently in a lot of documents and assign high weightage to terms that are important to specific documents as they can be the defining features or good predictors of a class. This is accomplished by taking the product of two metrics, that is, the number of times a word appears in a document, called term frequency, and the inverse document frequency of the word, which is a measure of whether a term is common or rare across the corpus of documents Zeng (2018). It uses a measure of how often the words appear in the documents, and the word count is weighted by that measure Kumar (2020).

For classification, recent works implement various machine learning and deep learning classification models including neural networks. Common machine learning models include Random Forest Classifier, Logistic Regression and XGBoost used in Gupta (2021), Naive Bayesian, KNN, *etc.* Anandh (2020) uses 7 machine learning algorithms with different component extraction methods to compare their results and found out that using TF-IDF feature extraction with Logistic Regression gave the best results producing an accuracy score of 86% and a logarithmic loss of 0.6812. Gupta (2021) uses three machine learning classification models, namely, Logistic Regression (LR), Random Forest (RF), and XGBoost (XGB), each with three text transformation models, namely, CountVectorizer, TfIdfVectorizer and Word2Vec, it results in accuracy scores between 38% and 49%, the best among them being XGBoost with CountVectorizer which gives an accuracy score of 48.49%.

Most researchers have selected a single classification model or its variations with large feature sets as the research articles in the datasets are very lengthy, with some articles even consisting of more than 80,000 words. However, very limited amount of work use feature selection and dimensionality reduction techniques to reduce the feature set in order to make the classification process more efficient. Low accuracy scores could be the lack of use of efficient text transformation and dimension reduction models. Xu (2019) uses metrics like extracting the top 2500 tf-idf features of each ID, sorting them according to importance and then using the top 500 sorted words to replace the texts to reduce the feature set, as the performance of ML models is dependent on feature extraction methods. Other works that use deep learning models and neural networks rely on the model’s automatic acquisition of feature expression capabilities, thus eliminating complex manual feature engineering processes and reducing possible application costs Su (2020).

The main contribution is related to identifying text features with the relevant gene mutations. We apply an innovative combination of textual feature selection using TF*IDF, which is further reduced through semantic text feature selection from SVD to address large, sparse textual feature dimensions. Identifying textual features from relevant medical literature that can play a vital role in gene mutation which eventually helps in accurately classifying cancer genes is regarded as our main contribution in the research realm.

## 3 Materials and methods

### 3.1 Approach

**FIGURE 1:**
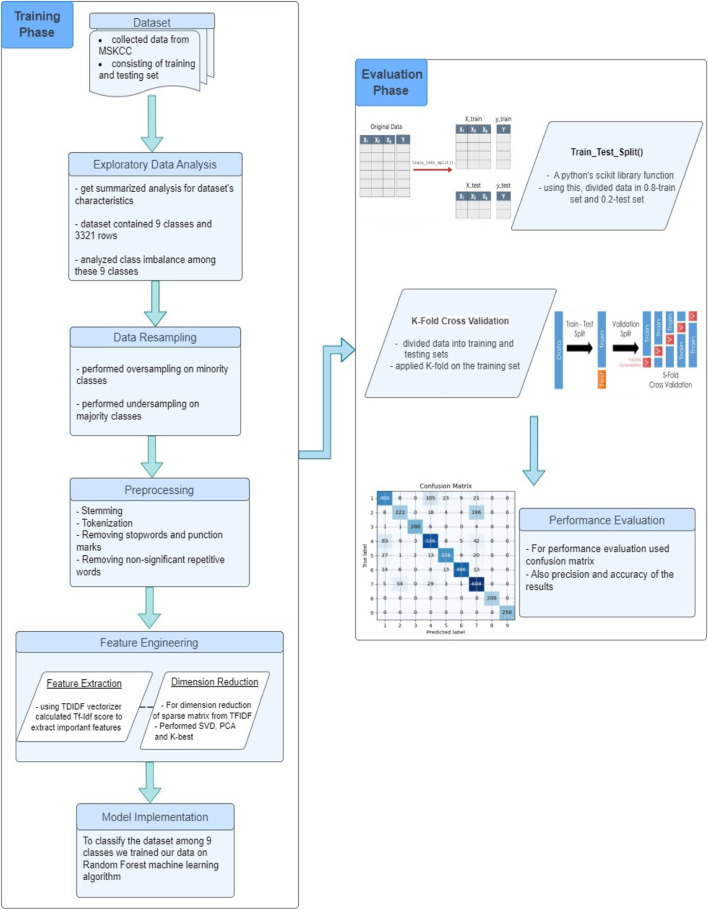
The systematic working flow of the research procedure.

The detailed pipeline of our proposed model is illustrated in Figure 1. We initiated our study by acquiring data from the Memorial Sloan Kettering Cancer Center (MSKCC), which was made available through a Kaggle competition. There were 9 mutation classes, but the data was not class-balanced, therefore we performed oversampling and undersampling to achieve a balanced distribution across classes. We then performed some preprocessing steps like stemming, tokenization, and the removal of stopwords and non-significant repetitive words. Further, we adopted TF-IDF algorithm to quantify the significance of each term based on its frequency across the entire collection of documents. To further refine the dataset, we explored multiple dimension reduction techniques including SelectKBest, PCA and SVD, and applied SVD to reduce the feature set and generate the final input matrix due to its effectiveness. In the last stage, we experimented with different classification models, including Logistic Regression and Random Forest, using Naive Bayes as a baseline model. The Random Forest model outperformed the rest, showing superior accuracy and precision. We trained our Random Forest model on the input matrix and evaluated our approach based on accuracy and precision measures. The following sections will delve into the details of each step in the process.

### 3.2 Dataset

The dataset for this research work is obtained from Kaggle provided by the Memorial Sloan Kettering Cancer Center (MSKCC) where various world-class researchers and oncologists have contributed to its preparation. The MSKCC dataset consists of training and testing datasets of gene mutations along with textual clinical evidences. Genetic mutations provided in the training set are manually annotated by clinical experts and are classified into 9 different classes as shown in Table 1 after interpreting the text-based clinical evidences. To gain a deeper understanding into the MSKCC dataset, we first perform exploratory data analysis (EDA) in Python to summarize the main characteristics of the data and projected them with visual representations such as tables and graphs Pramanik (2019). It was discovered that the class distribution of samples in the training dataset was highly imbalanced.

**TABLE 1:** Class labels with their descriptions.

Class label	Description
1	Gain-of-function
2	Likely-Gain-of-function
3	Loss-of-function
4	Likely Loss-of-function
5	Neutral
6	Likely Neutral
7	Switch-of-function
8	Likely Switch-of-function
9	Inconclusive

**FIGURE 2:**
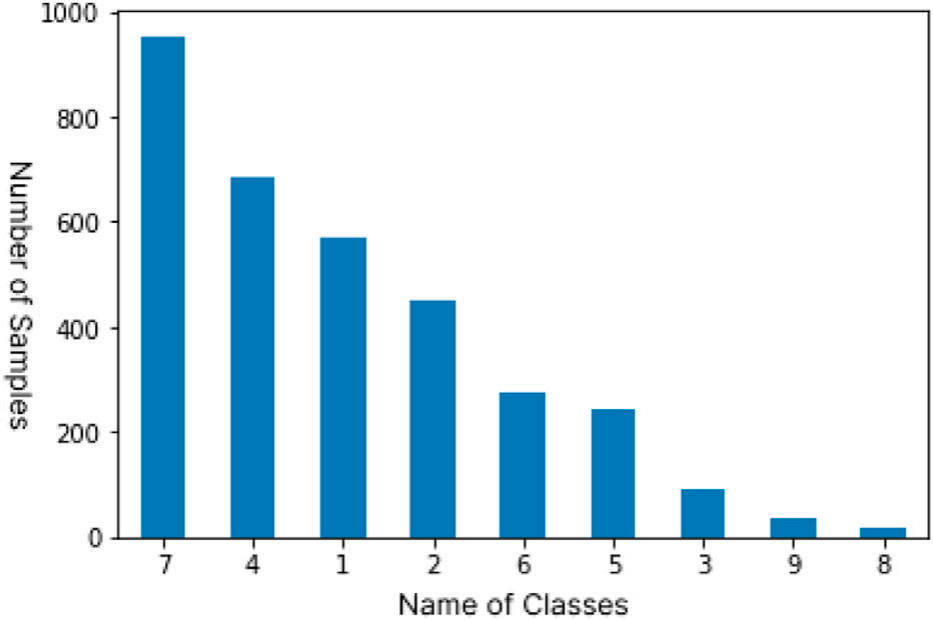
Distribution of samples among the 9 classes in the original data set.

As shown in Figure 2, the distribution of samples in the classes are highly imbalanced which is a major challenge in the dataset. Studies have shown that imbalanced data can majorly affect the meaning of accuracy Luque (2019). Imbalanced data is characterized by having more instances belonging to one class than others. It is a very common trait and it can be observed in many real world problems. As shown in the bar graph, classes 3,8 and 9 have very few instances of the mutations, the lowest being 19 samples for class 8 which is just 0.6% of the total number of samples, while class 7 hosts 952 instances which makes up 28.7% of the total and is the most common class in the dataset. Since the instances belonging to the minority class(es) rarely occur, the rules for classifying these classes tend to be rare, undiscovered, or ignored Ferreira (2019),Kraiem (2021), hence classification for the minority classes could be largely inaccurate. This issue can be practically resolved by oversampling and undersampling of the data to closely balance the dataset Bruno (2019). Experimental results from Zeng (2018) show that using oversampling and undersampling methods on imbalanced datasets significantly improve the accuracy for the minority classes. We use resampling on the training dataset by using a combination of oversampling and undersampling to increase the number of samples in minority classes and reduce samples in majority classes, respectively, using Python’s imblearn library which brings about an improved balance of samples in classes of our dataset.

### 3.3 Preprocessing

For preprocessing of data, we filter stop words like “the”, “is”, “in”, “of” and remove punctuation marks from text to reduce redundancy. In addition to NLTK’s English stop words and punctuation, unimportant keywords by observing the text, for example, words shown in Table 2 are also removed. We further removed words that occurred very frequently in each of the 9 classes and hence played no significant role in prediction of the classes. These methods considerably reduced the feature set which would make the classification process faster, more efficient and would cause it to use meaningful features for classification. Stemming is also performed after removing stop words and punctuation marks to categorize words like “cell” and “cells”, “mutate”, “mutates”, “mutated”, mutation” and “mutations” into a single category.

**TABLE 2:** List of unnecessary removed words.

Unnecessary Words
fig, figure, table, method, result, using, conclusion, author, find, found, demonstrate, may, done, however, although

### 3.4 Feature engineering

Due to the nature of the given dataset being text-based, conversion of text features, which are categorical data, to numeric data is needed for any classification algorithms to be adopted. We used the TF-IDF feature extraction and conversion method to quantify words from the text corpus using TfidfVectorizer from Python’s scikit-learn library. In TfidfVectorizer, the overall weightage of a word occurring in a document is considered Kumar (2020). Through this, we can penalize the words that occur most frequently in a lot of documents and assign high weightage to terms that are important to specific documents as they can be the defining features or good predictors of a class. This is accomplished by taking the product of two metrics, that is, the number of times a word appears in a document, called term frequency, and the inverse document frequency of the word, which is a measure of whether a term is common or rare across the corpus of documents Bernard (2022). It uses a measure of how often the words appear in the documents, and the word count is weighted by that measure Kumar (2020).

To further reduce the dimension of the sparse matrix obtained by the TF-IDF vectorizer, different feature selection, and dimensionality reduction techniques can be used to select the features that are most important for the classification of mutations and to create projections of the high-dimensional features onto a fewer-dimensional space. We explored different feature extraction and dimensionality reduction methods like Principle Component Analysis (PCA), Singular Value Decomposition (SVD) and k-best features and evaluated the effectiveness of the extracted features against classifiers. We found out that SVD performed best compared to the other techniques.

### 3.5 Model implementation

After feature engineering, the final stage of our pipeline involved training a classification model on the processed input matrix. In our pursuit of model selection, we explored various algorithms, including Naive Bayes, Logistic Regression and Random Forest models. After comparison of these classifiers based on their accuracies, we discovered that the Random Forest model was the most effective in classifying the mutations among the rest. Therefore, we applied the versatile Random Forest algorithm to address the challenging task of classifying textual evidences. It is proven to be highly effective ensemble learning method where predictions are made by aggregating the outputs of multiple decision trees. It is not only a robust and accurate model but also handles high-dimensional data, less prone to over-fitting, and provides interpretability. We divided the dataset into subsets for training and testing. Utilizing the training data, we trained the Random Forest model while utilizing cross-validation to improve the hyper parameters. With each decision tree trained on a separate subset of characteristics and data samples, the Random Forest algorithm created an ensemble of decision trees.

## 4 Testing and evaluation

To assess the performance of the model, we used train_test_split() function and the k-fold cross validation. The train_test_split() is a function in Python’s scikit-learn library used to split the available data into training and testing sets. The test size is set to 0.2, which means that 20 percent of the data will be used for testing and the remaining 80 percent will be used for training. The stratified parameter is set to ensure that the class distribution in the training and testing sets is similar. This is important to avoid bias in the model evaluation, especially when dealing with imbalanced datasets, where one class may have significantly more samples than the other. When the stratified parameter is set, the split is done in such a way that the proportion of each class in the dataset is preserved in both the training and testing sets. For the k-fold cross validation the dataset was divided into testing sets and was applied on the training set with the cv parameter is set to StratifiedKFold (n_splits = 10, shuffle = True, random_state = 1). This means that the training data is divided into 10 subsets of equal size, and each subset is used as a testing set while the remaining 9 subsets are used to train the model. This process is repeated 10 times using stratified sampling to preserve the class distribution in each fold. The shuffle parameter is set to True to shuffle the dataset before splitting, and the random_state parameter is set to 1 to ensure that the same random shuffling is applied every time the function is called.

## 5 Results

Table 3 presents the percentage accuracies of the classification models explored in our study, with Naive Bayes employed as a baseline. The table also shows the effects of utilizing Singular Value Decomposition (SVD) as a feature reduction technique, as well as the impact of employing oversampling and undersampling data. The results reveal that the Random Forest model, when combined with SVD and resampling techniques, exhibited the highest performance among the models considered. This combination proved to be particularly effective in enhancing our classification accuracy.

**TABLE 3:** Results of different combinations.

Classification algorithm	Average accuracy (%)
Naive Bayes	58.5
Random Forest	65.81
Logistic Regression	66.86
Logistic regression with SVD	67.8
Random Forest with SVD	70.1
Random Forest with SVD after Resampling	81.8

**FIGURE 3:**
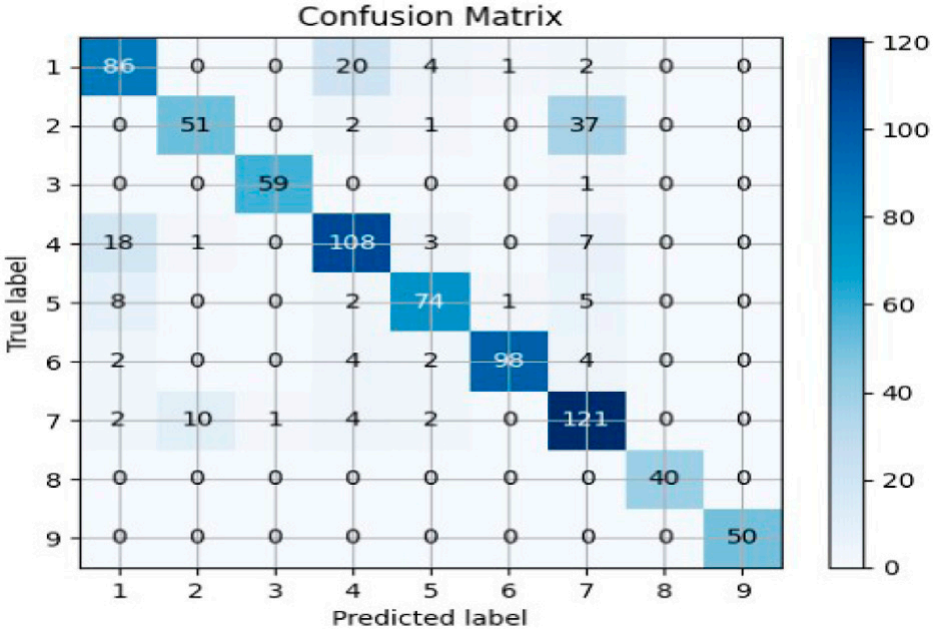
Confusion matrix for Random Forest applied with SVD feature reduction.

The Random Forest model was applied to a dataset using TF-IDF and truncated SVD for feature extraction. After testing the model using both of Python’s train_test_split and cross_val_predict functions, an accuracy of approximately 82% was observed. The obtained accuracy indicates that the model was able to accurately classify the text data with a high degree of precision. This result suggests that the combination of TF-IDF and truncated SVD is a powerful feature extraction technique for text classification tasks. Through investigating several feature selection strategies, text transformation models, and classification models, we used a model that combines the techniques that perform the best on the test data. The performance of the classifiers is evaluated using a confusion matrix shown in Figure 3 and a classification report shown in Table 4. The confusion matrix shows the number of true positives, true negatives, false positives, and false negatives, while the classification report displays various metrics such as accuracy score, precision, recall, and f1 measure. These metrics provide a comprehensive view of the performance of the classifiers and can be used to compare different models and techniques.

**TABLE 4:** The table presents the classification report for the developed classifier model which provides valuable insights into the model’s performance. It includes important performance metrics such as precision, recall, F1 measure, and support. Precision represents the accuracy of positive predictions, recall indicates the ability to identify positive cases correctly, and the F1 measure combines precision and recall into a single metric. The support denotes the number of samples in each class.

Class	Precision	Recall	F1-score	Support
1	0.75	0.77	0.76	113
2	0.87	0.49	0.63	91
3	0.98	1.00	0.99	60
4	0.73	0.77	0.75	137
5	0.87	0.81	0.84	90
6	0.93	0.87	0.90	110
7	0.69	0.89	0.78	140
8	1.00	1.00	1.00	40
9	0.98	1.00	0.99	50
accuracy			0.82	831
macro avg	0.87	0.84	0.85	831
weighted avg	0.83	0.82	0.82	831

Our proposed model tries to learn the implicit correlation between genetic mutation and its variant with the textual description available to it, through supervised learning. In our test data, we have found the following genes and mutations related to some of the known target classes. Here, Tables 5, 6 presents classification results of a few genetic mutations generated by our trained model from the test set and the validation set.

**TABLE 5:** Test set.

ID	Gene	Variant	Class
0	ACSL4	R570S	7
121	BRCA1	T1773I	1
215	BRCA2	G173C	1
562	TP53	E180K	4
3607	ABCB11	R432T	4

**TABLE 6:** Validation set.

ID	Gene	Variant	Class
3141	KRAS	T58I	7
3182	AKT3	Fusions	2
3183	TCF3	N551K	4
3281	RET	C634W	2
3302	RUNX1	R166Q	4

## 6 Comparison with other models

Based on the results presented in Table 7, it can be concluded that our proposed model outperformed the other models in terms of predictive performance on the Kaggle dataset by achieving the highest F1 scores using both 10-fold cross-validations.

**TABLE 7:** Comparison with other models.

Research work	Model	Accuracy (%)	Remarks
Waykole (2018)	Logistic Regression with TF-IDF	64.0	Poor accuracy, only text features used
Xu (2019)	CNN	64.8	Poor accuracy, only text features used
Smagulove (2019)	LSTM	74.3	Poor accuracy, only text features used
Gupta (2021)	Logistic Regression with Count Vectorizer	38.15	Poor accuracy, only text features, class imbalance
Gupta (2021)	Random Forest with Countvectorizer	47.47	Poor accuracy, only text features, class imbalance
Gupta (2021)	XGB with Countvectorizer	48.49	Poor accuracy, only text features, class imbalance
Gupta (2021)	Logistic Regression with TF-IDF	38.54	Poor accuracy, only text features, class imbalance
Gupta (2021)	Random Forest with TF-IDF	48.28	Poor accuracy, only text features, class imbalance
Gupta (2021)	XGB with TF-IDF	49.73	Poor accuracy, only text features, class imbalance
Gupta (2021)	Logistic Regression with Word2Vec	46.71	Poor accuracy, only text features, class imbalance
Gupta (2021)	Random Forest with Word2Vec	45.02	Poor accuracy, only text features, class imbalance
Gupta (2021)	XGB with Word2Vec	48.22	Poor accuracy, only text features, class imbalance
Gupta (2021)	RNN	70.78	Poor accuracy, only text features, class imbalance
This work	Random Forest with TF-IDF and SVD	82.0	Outperforms existing models

Deep learning models have been used less commonly but include Recurrent Neural Network (RNN) used in Gupta (2021) which gives an accuracy score of 71%, a combination of Convolutional Neural Network (CNN) and Bidirectional Gated Recurrent Unit (BiGRU) in Xu (2019) giving an accuracy of 84.4%, and Bidirectional Encoder Representations from Transformers (BERT) used in Su (2020) after fine-tuning have a logarithmic loss of 0.8074. Gupta (2021) implements the Recurrent Neural Network (RNN) model of deep learning with CountVectorizer, TfIdfVectorizer, and Word2Vec giving an accuracy of 71% with pre-trained Word2Vec which is the highest in that paper. The BERT + abstract truncation method used in Su (2020) has given a performance with 0.8074 logarithmic loss and 0.6837 recall. The 0.705 F-measure score is limited by the extreme shortage of training data.

## 7 Discussion

The latest studies suggest that clinically actionable medications play a critical role in cancer treatment. And to prepare these medicines pathologists, oncologists, and medical staff spend plenty of time analyzing the cancer reports manually. Considering this time-consuming process, our study aimed to modernize this process and provide ease to medical professionals in analyzing clinical evidence technically. For this, we have used random forest, a machine algorithm that classifies all clinical evidence into 9 different classes with an accuracy of 81.05% by which oncologists can identify the specific class for a genetic mutation. Conclusively, our study adds value to the classification process of clinical evidences as it replaces the manual process with a technically intelligent process giving 81.05% accurate results which can be useful in developing actionable medication for cancer patients.

In order to enhance user accessibility and practicality, the developed classifier model was seamlessly integrated with a web interface. This integration allows users, particularly oncologists, to easily input test data and obtain real-time results. The web interface provides a user-friendly and intuitive platform where users can conveniently enter the genetic mutation and its corresponding textual evidence. The system then processes the input through the trained classifier model and promptly generates the output class label.

**FIGURE 4:**
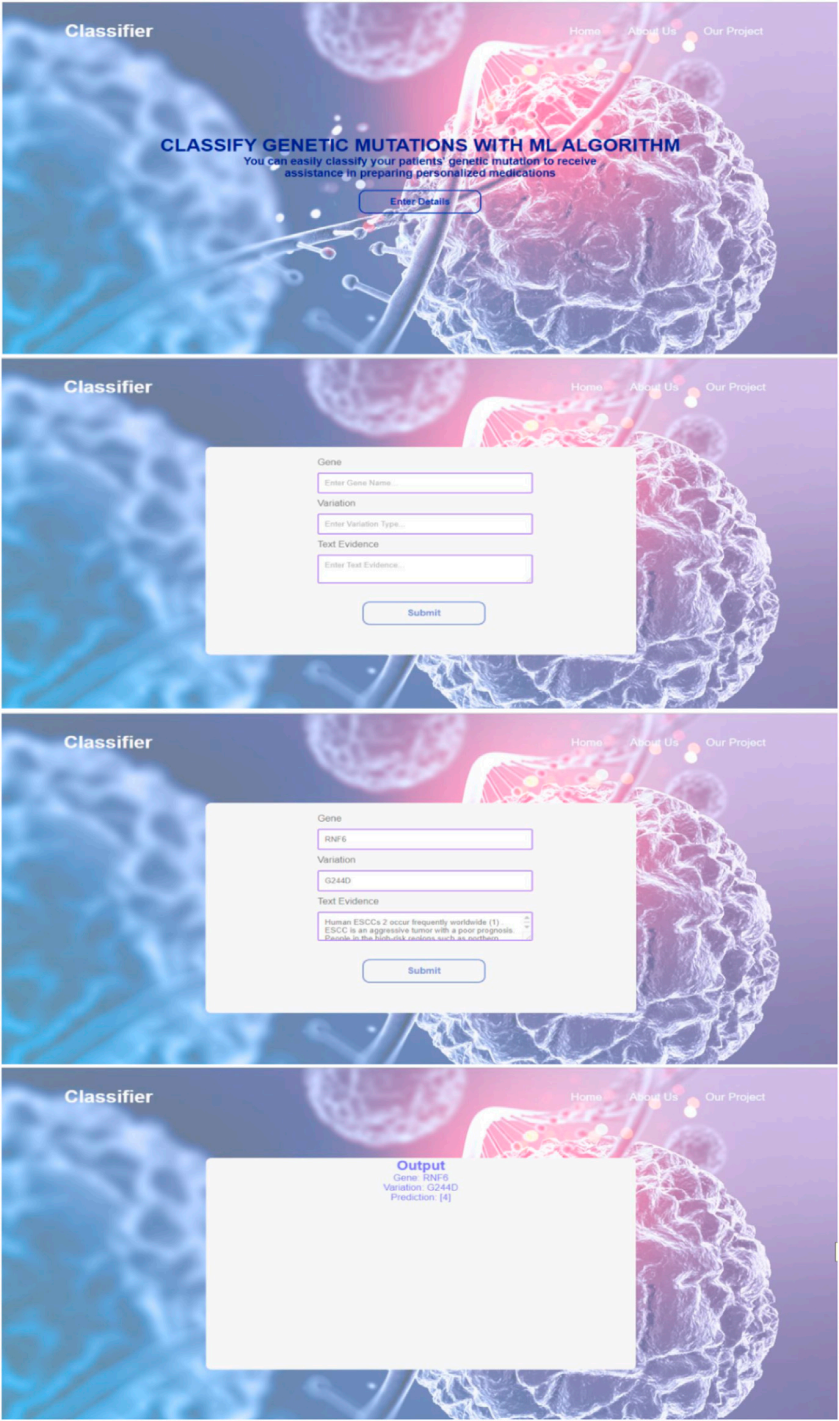
Screenshots from the web interface integrated with the model.

The integration of the model with the web interface streamlines the workflow and eliminates the need for manual implementation or technical expertise. By incorporating visual representations and interactive elements, the web interface enhances the overall user experience, enabling efficient utilization of the classifier model. Additionally, the web interface facilitates quick verification and validation of the results, empowering users to make informed decisions based on the predicted classifications. Figure 4 are the screenshots that visually demonstrate the functionality and usability of the web interface, providing concrete evidence of its practical application in the field of personalized cancer treatment.

## 8 Limitation of the study

One of the major limitations of this work is the availability of the data that is used to identify the gene. The current data is from the research papers till 2017. There is rapid growth in the oncological gene identification research and a lot of work has been done very recently. It is pertinent to know that if all this data is available to process by our proposed model we will be able to identify a lot of active gene mutations and it will enable us to know more about these changes. Moreover, clinical validation is also very important and it is also very time-consuming. We believe that our automation of classification, classified genes, and relevant text-feature descriptions makes it easy for human experts to verify the same.

## 9 Conclusion

This research work is carried out to propose a multi-class classifier to classify genetic mutations into one of the nine classes based on the textual description of the genetic mutations provided as clinical evidence for classification. The proposed system can greatly facilitate oncologists in the classification of genetic mutations, reducing the burden of manual work and enabling a more efficient and accurate adoption of personalized medicine. Through a user-friendly web interface, oncologists can simply input the genetic mutation, its variant, and textual evidence, into the system. With just a few clicks, the system automatically generates the appropriate class label, enabling oncologists to verify their answers easily. This advancement in technology has the potential to revolutionize cancer treatment, improving patient outcomes and contributing to the progression of personalized medicine.

## Data Availability

The raw data supporting the conclusions of this article will be made available by the authors, without undue reservation.
